# Nondestructive Measurement of Hemoglobin in Blood Bags Based on Multi-Pathlength VIS-NIR Spectroscopy

**DOI:** 10.1038/s41598-018-20547-2

**Published:** 2018-02-02

**Authors:** Shengzhao Zhang, Gang Li, Jiexi Wang, Donggen Wang, Ying Han, Hui Cao, Ling Lin

**Affiliations:** 1 State Key Laboratory of Precision Measurement Technology and Instruments, Tianjin University, Tianjin, 300072 China; 2 Tianjin Key Laboratory of Biomedical Detecting Techniques and Instruments, Tianjin University, Tianjin, 300072 China; 3 Beijing Institute of Blood Transfusion Medicine, Academy of Military Medical Sciences, Beijing, China

## Abstract

Hemoglobin concentration is an indicator for assessing blood product quality. To measure hemoglobin concentration in blood products without damaging blood bags, we proposed a method based on visible-near infrared transmission spectroscopy. Complex optical properties of blood bag walls result in measurement irregularities. Analyses showed that the slope of the light intensity-pathlength curve was more robust to the influence of the blood bag wall. In this study, the transmission spectra of red blood cell suspensions at multiple optical pathlengths were obtained, and the slopes of logarithmic light intensity-pathlength curves were calculated through curve fitting. A nondestructive measurement of hemoglobin content was achieved by using a regression model correlating slope spectra and hemoglobin concentration. Sixty samples with hemoglobin concentrations ranging from 72 to 161 g/L were prepared. Among them, 40 samples were used as a calibration set, and the remaining 20 samples were used as a prediction set. The determination coefficient of the prediction set was 0.97, with a mean square error of 2.78 g/L. This result demonstrates that a non-destructive measurement of hemoglobin levels in blood bags can be achieved by multiple-pathlength transmission spectroscopy.

## Introduction

According to Chinese national standard quality requirements for whole blood and blood components (GB18469-2012), hemoglobin concentration in blood must be greater than 100 g/L^1^. To ensure the quality of blood products, the concentration of hemoglobin must be determined. Conventional chemical testing methods would require opening the blood bag and would thus waste the blood because the remainder of the blood in the opened bags not used for testing cannot be used for blood transfusion and must be discarded^2,3^. Visible-near infrared spectroscopy has been widely used for non-invasive analyses of compositions. This method is more efficient, environmentally friendly, and economical than other chemical analysis methods. It has been applied in medicine, chemical industry, food, agriculture, and other fields^4–9^ and has also been used for the non-invasive determination of hemoglobin concentration or free hemoglobin concentration^2,3,10–14^. Yan Wang proposed an *in vivo* hemoglobin detection method called Dynamic Spectrum^15^. A. Opp designed a sensor for the non-invasive determination of hemoglobin in sampling tubes^13^. Linna Zhang also proposed a method for hemoglobin concentration determination based on near infrared transmission spectroscopy^16^. Ling Lin attempted to detect free hemoglobin in blood products using transmission and fluorescence spectroscopies^3^. The results reported in those studies show the feasibility of detecting hemoglobin non-invasively based on optical methods. However, most studies on hemoglobin detection have been performed in sample tubes or cuvettes, whereas nondestructive hemoglobin measurements in blood bags have rarely been reported. Unlike cuvettes or sample tubes, blood bags are made of flexible materials and have variable shapes. Several factors have to be considered when designing a device for performing in-blood bag measurements of hemoglobin levels because the pathlength of transmitted light must be consistent. Light intensity losses due to reflection and attenuation are irregular for blood bags and may cause errors if the methods are not carefully designed.

In this study, we designed a device to obtain transmission spectra over multiple pathlengths of blood in blood bags. Using data processing, the effects of the flexible nature of the bags on the optical signals were eliminated. We developed a nondestructive measurement method for determining hemoglobin concentrations using a chemometric tool without opening the blood bag.

## Influence of the Wall of Blood bag

**Figure 1:**
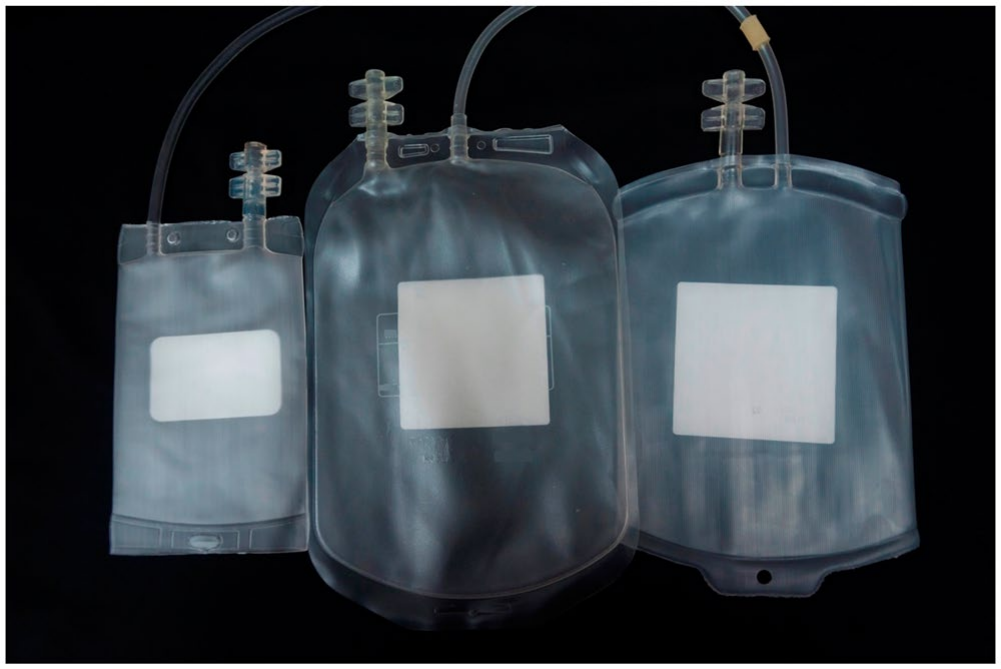
Blood bags used for blood collection and transfusion.

**Figure 2:**
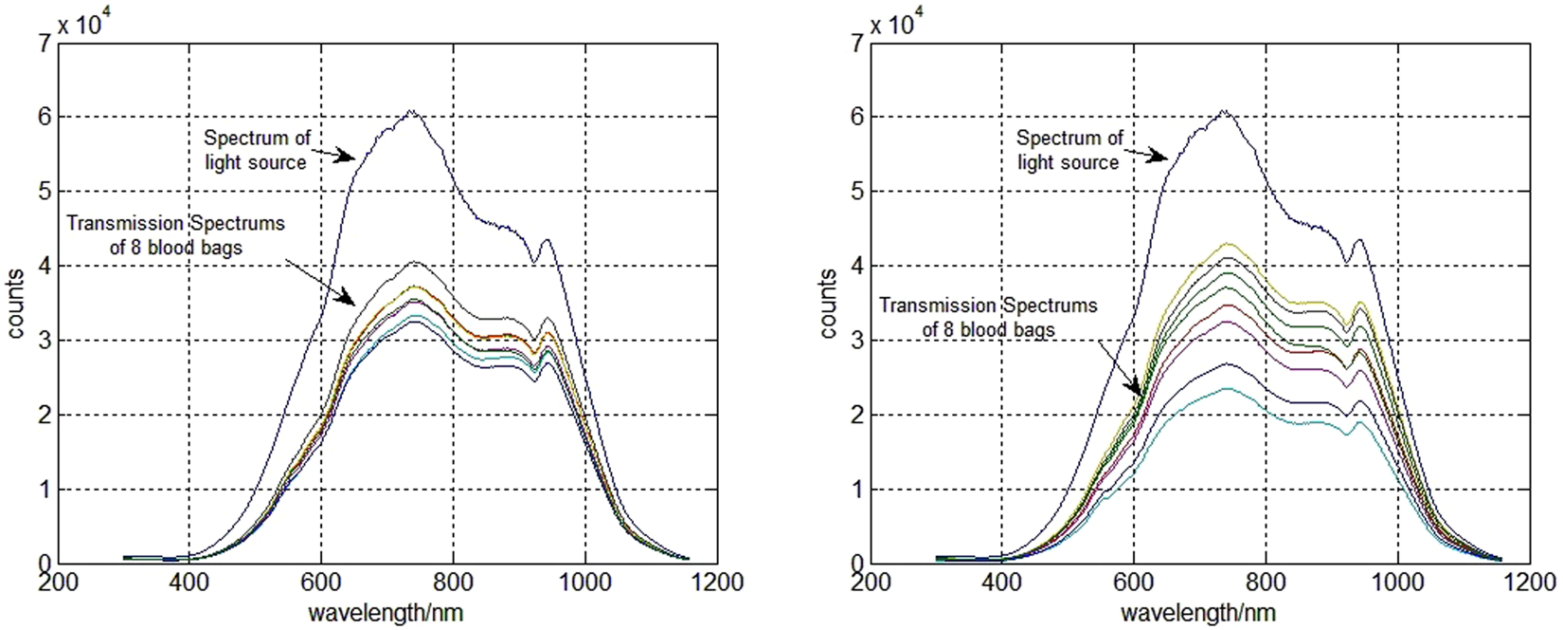
(**a**) The spectrum of the light source and the transmission spectra of 8 empty blood bags manufactured by Weigaogroup Co. Ltd. (**b**) The spectrum of the light source and the transmission spectra of 8 empty blood bags manufactured by Nigale Biological Technology Co. Ltd.

Blood bags are used for storing bloods. In Fig. 1, blood bags of 400 ml, 200 ml and 100 ml are displayed. Blood bags are made of polyvinylchloride (PVC), which is compatible with red blood cells and permeable to gases, such as oxygen and carbon dioxide^12^. However, this material is non-uniform, is heterogeneous in structure, has highly irregular scattering properties, and has no consistent optical surface. To investigate the characteristics of the blood bags, we measured the transmission spectra of 16 empty blood bags manufactured by two different companies. Among the 16 blood bags, 8 were from a batch manufactured by Weigaogroup Co. Ltd., and 8 were from a batch manufactured by Nigale Biological Technology Co. Ltd. The spectrum of the light source used in the measurements and the transmission spectra of the 16 empty blood bags are shown in Fig. 2. Light transmission through the bags was attenuated by approximately 40% to 60%, and spectral changes were also observed. The transmission spectra of different empty blood bags are different, even between blood bags from same company.

## Simulation Study and Data Processing Strategy

### Simulation Study

**Figure 3:**
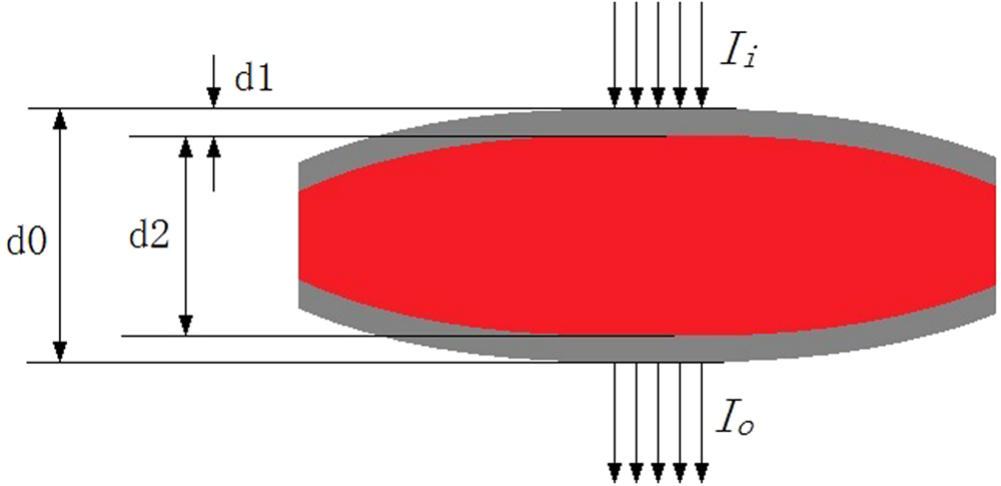
Schematic of light passing through a bag and the liquid sample inside.

The wall thicknesses of the blood bags varied from 0.5 mm to 0.7 mm between the different batches, and the optical characteristics of blood varied with different blood bags. While the total thickness of the blood bag and the contained blood is relatively easy to determine, the exact thickness of the contained blood is difficult to measure because of variations in the blood bag wall thickness. This wall thickness variation along with wall optical property variations have confounding effects on the optical signal. Figure 3 shows a beam of light passing through a blood bag with red blood cell suspension inside. According to the Modified Lambert-Beer’s Law (MBML), when the intensity of the incident light at a wavelength λ is ${I}_{i}^{\lambda }$, the intensity ${I}_{o}^{\lambda }$ of the transmitted light beam can be expressed by Eq. (1), where ${\varepsilon }^{\lambda }$ is the molar extinction coefficient of the blood components, *c* is the concentration of the hemoglobin to be measured, *d0* is the total thickness of the blood bag, and *d1* is the thickness of the wall of the blood bag itself. ${B}^{\lambda }$ is a path factor, which is used to describe the lengthening of the propagation path of light due to scattering of the object. *G* represents the unknown loss of the light intensity caused by the wall of blood bag.

1 $$\mathrm{ln}({I}_{o}^{\lambda })=\,\mathrm{ln}({I}_{i}^{\lambda })-{\varepsilon }^{\lambda }c(d0-2d1){B}^{\lambda }-G$$

Because blood bags are made of flexible materials, the optical signal can be measured at different total thicknesses by squeezing the blood bag. By changing the total thickness of the blood bag, the “changing part” of the optical signal is less dependent on the blood bag wall or blood thickness. The slope of logarithm of the light intensity to the thickness is approximately ${s}^{\lambda }={\varepsilon }^{\lambda }c{B}^{\lambda }$. To determine how the “changing part” resisted blood bag variations, simulations were conducted.

Monte Carlo simulations were performed. Our simulation program was based on Erik Alerstam’s CUDAMCML program^17^. The script simulates light passing through a package and the liquid sample inside the package, as shown in Fig. 3. The diameter of the incident light beam was set to 1 mm. The transmitted light is collected by an optical fiber probe with a diameter of 10 mm and a numerical aperture of 0.22. The optical parameters of the package and the liquid sample in the Monte Carlo simulations are listed in Table 1. In Table 1, *n*, μa, μs, *g* and *d* denote the refraction index, absorption coefficient, scattering coefficient, anisotropy factor and thickness, respectively. The absorption coefficients are approximately the same as those of 70–240 g/L hemoglobin at 800 nm^18^. The intensities of the light collected by the optical fiber probe were calculated.

**Table 1:** Optical parameters in the Monte Carlo simulation.

Package	Liquid Sample
*n*1 = 1.3	*n*2 = 1.4
μa1 = 0.005 cm^−1^	μa2 = 0.35, 0.40, …, 1.55 cm^−1^
μs1 = 75, 125, 175 cm^−1^	μs2 = 100 cm^−1^
*g* = 0.8	*g* = 0.75
*d*1 = 0.5, 0.7 mm	*d*2 = 3.0, 3.1, …, 5.0 mm

**Figure 4:**
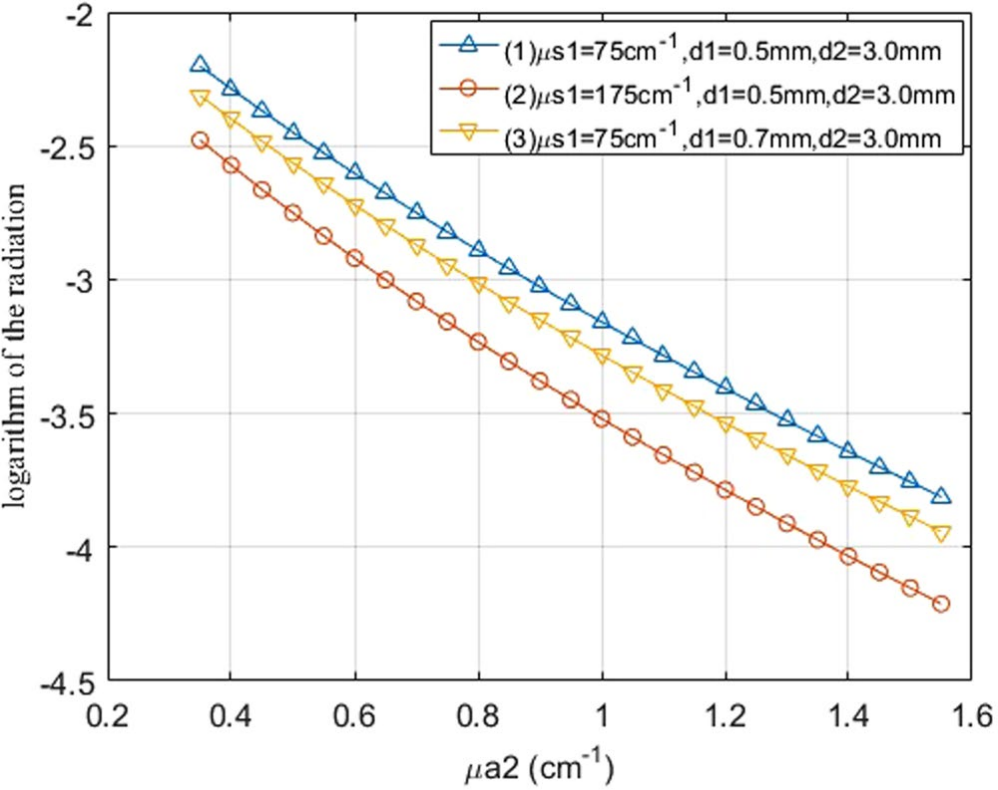
Logarithm of the transmitted light intensity with different package parameters for μa2 ranging from 0.35–1.55 cm^−1^.

The logarithm of the transmitted light intensity with different package parameters and the liquid sample absorption coefficients are shown in Fig. 4. The package thicknesses corresponding to the first and second curves are both 0.5 mm, whereas the scattering coefficients are different. The scattering coefficients of the package corresponding to the first and third curves are both 75 cm^−1^, whereas the thicknesses are different. This indicates that when the optical parameters or the package thickness change, the transmitted light intensity exhibits apparent differences. To quantitatively describe the differences between the two curves, a coefficient called “difference coefficients” (*DC*) is defined here, as is described in Eq. (2)^19^. This coefficient can be used to assess the degree of difference between two variables P and Q. In the equation, P and Q are written as *p*_*i*_ and *q*_*i*_. The numerator of the *DC* is squares of the difference of the two variables and the denominator is the product of the standard deviation of P and Q. When the variables are identical, the coefficients would be zeros. If the difference between P and Q are large, the *DC* will also be large. The *DC* of curve (1) and curve (2) in Fig. 4 is 12.00, and the *DC* of curve (1) and curve (3) is 1.56.

2 $${\rm{DC}}({\rm{P}},{\rm{Q}})==\frac{{\sum }_{{\rm{i}}}^{{\rm{n}}}{({{\rm{p}}}_{{\rm{i}}}-{{\rm{q}}}_{{\rm{i}}})}^{2}/{\rm{n}}}{\sqrt{{\sum }_{{\rm{i}}}^{{\rm{n}}}\frac{{({{\rm{p}}}_{{\rm{i}}}-\bar{{\rm{p}}})}^{2}}{{\rm{n}}}\ast {\sum }_{{\rm{i}}}^{{\rm{n}}}\frac{{({{\rm{q}}}_{{\rm{i}}}-\bar{{\rm{q}}})}^{2}}{{\rm{n}}}}}=\frac{{\sum }_{{\rm{i}}}^{{\rm{n}}}{({{\rm{p}}}_{{\rm{i}}}-{{\rm{q}}}_{{\rm{i}}})}^{2}}{\sqrt{{\sum }_{{\rm{i}}}^{{\rm{n}}}{({{\rm{p}}}_{{\rm{i}}}-\bar{{\rm{p}}})}^{2}{\sum }_{{\rm{i}}}^{{\rm{n}}}{({{\rm{q}}}_{{\rm{i}}}-\bar{{\rm{q}}})}^{2}}}$$

The logarithm of the light intensity was not linearly correlated with the thickness of the liquid sample because of scattering. We assumed that the logarithm of the light intensity was linearly correlated to the thickness over a small range.

**Figure 5:**
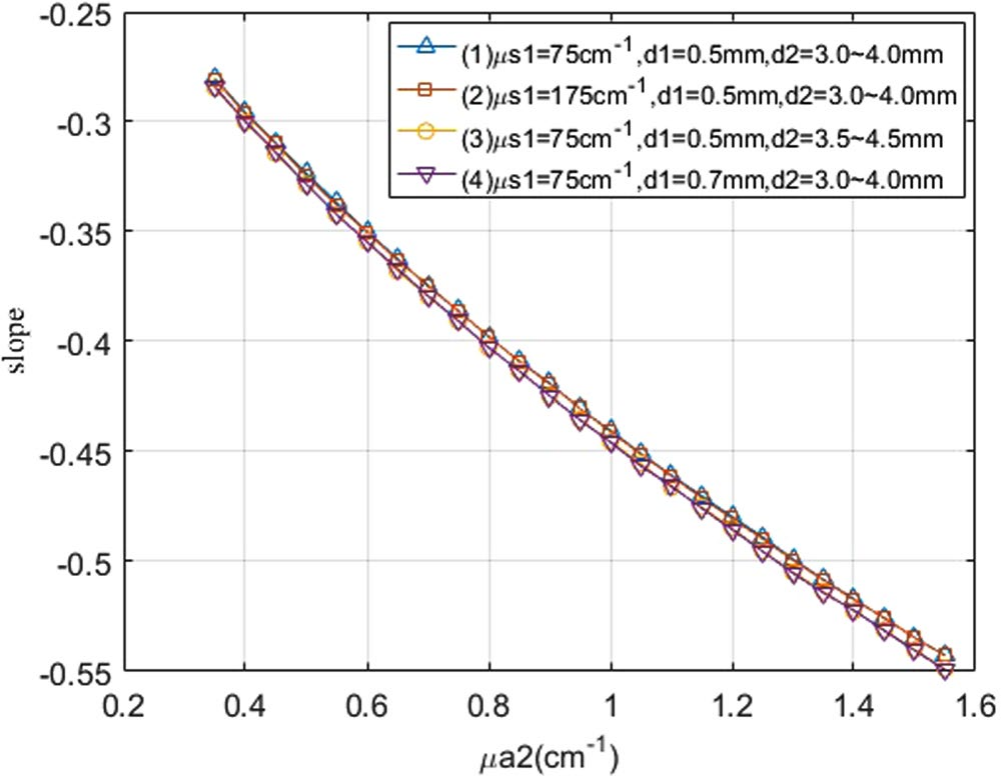
The slope of the logarithm of light intensity vs. sample thickness with different package parameters for μa2 ranging from 0.35–1.55 cm^−1^.

Based on this assumption, the slopes of the curves of the logarithm of the light intensity vs. thickness were obtained through linear fitting. Four slope-absorption coefficient curves are displayed in Fig. 5 for different package thicknesses, package scattering coefficients, and liquid sample thicknesses. In the legend, the “d2 = 3.0–4.0 mm” means the the slope was obtained when the thickness is from 3.0 mm to 4.0 mm. All curves approximately overlap. The *DC* between curve (1) and curve (2), between curve (1) and curve (3), and between curve (1) and curve (4) are 0.0012, 0.1059, and 0.1095, respectively. This indicates that variations in package thickness, package scattering, and sample thickness in this range can result in very small changes to the slopes. Because the slope changes with the sample absorption coefficients, the slope can be used to predict solute concentrations in liquid samples with fewer influences from the package.

### Data Processing Strategy

In most cases, a regression model that relates the transmission spectra and known solute concentrations is typically built first. To predict the unknown concentrations of a solution, the measured spectral data are input into the developed model. This method works well when the transmission spectra are measured from samples contained in a cuvette. However, because the optical properties and the thickness of the blood bags are highly variable, this model may not be suitable for the measurement of blood contained in a blood bag.

By squeezing the blood bag, the transmission spectra $\mathrm{ln}({I}_{o}^{\lambda })$ for multiple thicknesses can be obtained. If the spectra are measured for *l* = *l*_1_, *l*_2_,…,*l*_n_, spectra $[{I}_{o1}^{\lambda },{I}_{o2}^{\lambda }\ldots {I}_{on}^{\lambda }]$ are obtained. Using the least squares fitting method, the slope and intercept of the $\mathrm{ln}({I}_{o}^{\lambda })$ *vs l* curve can be obtained by making a one-order function curve fitting on $\mathrm{ln}({I}_{o}^{\lambda })$ and *l*. The arrangement of the slope of the curves at each wavelength forms a slope spectrum row vector. Partial least squares regression (PLSR) is a popular chemometric tool used to determine the fundamental relationship between two matrixes ***X*** and ***Y***.

3 $${\boldsymbol{Y}}={\boldsymbol{XB}}+{{\boldsymbol{B}}}_{0}$$

The results of the regressions are regression coefficient vectors ***B*** and ***B***_**0**_. In spectroscopic analytics, the independent variable ***X*** represents the spectral signals of samples, and the independent variable ***Y*** represents the quantitative data. In this paper, PLSR was used to develop a regression model to predict hemoglobin concentration in blood bags. Instead of using transmission spectra measured for single thicknesses, slope spectra were used as the independent variables in the regression.

## Materials and Methods

### Instrumentation

**Figure 6:**
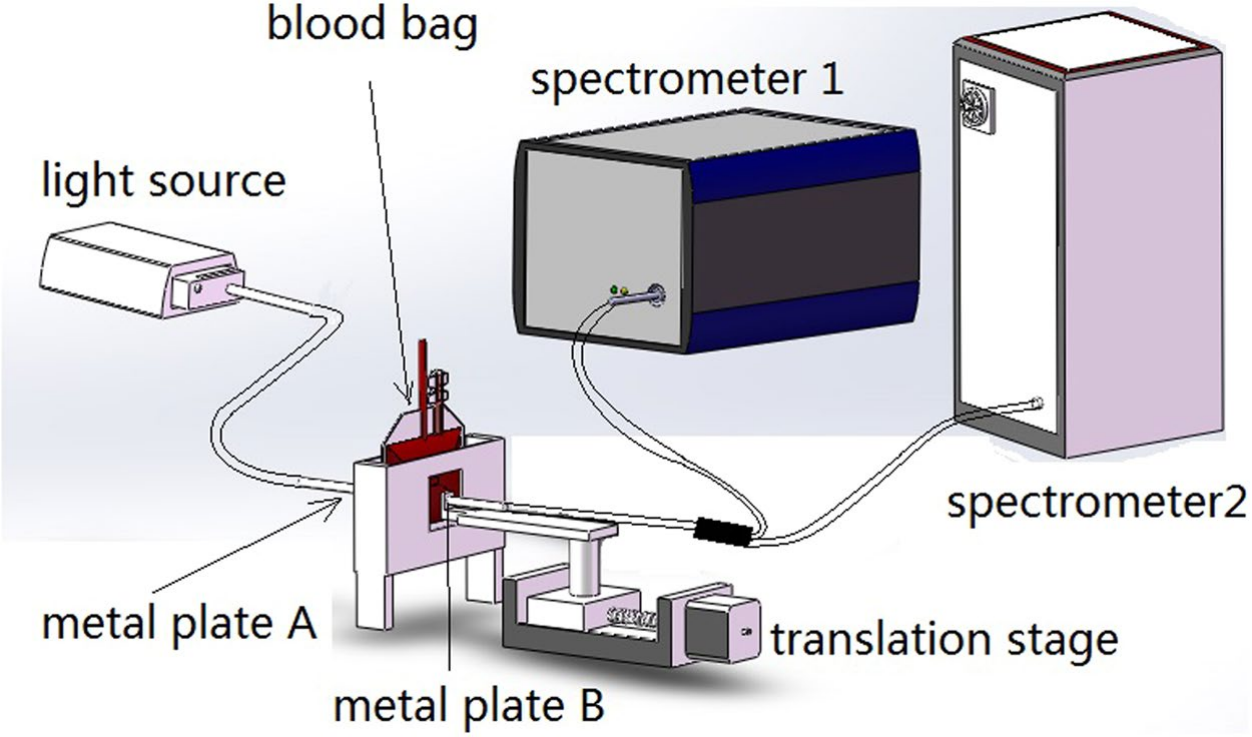
Schematic block Diagram of the hemoglobin detection device.

The spectral data acquisition setup is displayed in Fig. 6. The device comprised a light source, a spectrometer, a blood bag slot, optical fibers, an electrically controlled translation stage, and a computer. The device used a halogen light source (AvaLight-HAL-(S)-MINI, Avantes, Netherlands) with a wavelength range of 360–2500 nm. The output light power from the 600 μm optical fiber was 4.5 mW. Blood bag samples were placed in the blood bag slot between two metal plates. Both metal plates (A and B) had small holes and SMA 905 optical fiber interfaces. The halogen light source was connected to plate A through an optical fiber, and the diameter of the small hole in plate A was 2 mm. The area of plate B was smaller than that of plate A, and plate B was movable through a connection to an electrically controlled translation stage (TSA50-C, Zolix Instruments, China) with a repeat positioning accuracy of less than 5 μm and a resolution of 0.625 μm. The electrically controlled translation state could move plate B back and forth, thereby squeezing the blood bag to different thicknesses. A one-to-two fiber with a numerical aperture of 0.22 was connected to the moving metal plate to collect the transmitted light. The other two ends of the fiber were coupled to two spectrometers (AvaSpec-HS1024*58TEC-USB2 and AvaSpec-NIR256-1.7TEC, Avantes). The wavelength ranges of the spectrometers were 299–1160 nm and 1041–1772 nm, respectively. The numbers of wavelengths were 945 and 256 for the spectrometers.

### Samples

Sixty different hemoglobin (HB) concentrations of red blood cell suspensions contained in 100-ml standard PVC blood bags were provided by the Institute of Transfusion Medicine of the Academy of Military Medical Sciences. HB concentrations ranged from 72 to 161 g/L (measured by a hematology analyzer), as listed in Table 2. All experiments performed were in compliance with relevant laws and with the guidelines of the Chinese Academy of Sciences, Institute of Transfusion Medicine of the Academy of Military Medical Sciences and State Key Laboratory of Precision Measurement Technology and Instruments of Tianjin University. All mentioned institutes approved the experiments. The work described in this paper was carried out in accordance with the code of Ethics of the World Medical Association (Declaration of Helsinki) for experiments involving humans. People who donated blood gave their informed consent to the study participation and publication of identifying information. All blood bags were refrigerated at a temperature of 4 °C and were shaken and tested immediately after removal from the refrigerator. After each test, HB concentrations were detected using a hematology analyzer (BECKMAN COULTER AcT diff).

**Table 2:** HB concentrations in 60 blood samples.

No.	Hb (g/L)	No.	Hb (g/L)	No.	Hb (g/L)
1	114	21	110	41	128
2	144	22	131	42	134
3	123	23	154	43	107
4	114	24	130	44	108
5	111	25	128	45	123
6	146	26	137	46	142
7	135	27	146	47	154
8	102	28	133	48	128
9	127	29	136	49	134
10	132	30	120	50	124
11	116	31	141	51	129
12	116	32	126	52	135
13	101	33	118	53	137
14	151	34	128	54	126
15	143	35	98	55	110
16	118	36	133	56	122
17	106	37	72	57	150
18	125	38	88	58	115
19	161	39	82	59	94
20	114	40	105	60	83

### Experimental procedure

Multiple-pathlength transmission spectra of red blood cell suspensions in blood bags were collected using the above-described device. The halogen light source was turned on for at least 20 minutes to warm up before the measurements. A blood bag was placed between the two metal plates. All tests were conducted on the same blood bag. Transmitted light was collected by optical fiber and the two spectrometers; data were saved using AvaSoft 7.6 software. For each measurement, the integration time was adjusted so that the spectral peak reached at least 75% of the spectrometer range. At each measurement position, the spectral range was scanned 100 times, and the mean spectrum of the 100 scans was used for analysis. During the measurements, the electronically controlled translation stage shifted the metal plate to change the thickness the blood bag. Two different measurements were performed for each sample.

Measurement 1: we collected transmission spectra of blood bags at distances of 4.2 mm, 3.8 mm, 3.4 mm and 3.0 mm between the plates.

Measurement 2: we attached a PVC plastic film with a thickness of 0.3 mm to the blood bag and collected the transmission spectra at distances of 4.2 mm, 3.8 mm, 3.4 mm and 3.0 mm between the plates.

### Data Processing

Spectral data from 620 nm to 1772 nm were processed. The spectral data of a sample in each measurement were arranged as a 4*860 array as shown in array (4). The data in the first, second, third and fourth lines were measured when the distances between the plates were 4.2 mm, 3.8 mm, 3.4 mm and 3.0 mm, respectively.

4 $$[\begin{array}{cc}\begin{array}{cc}{I}_{1}^{\lambda 1} & {I}_{1}^{\lambda 2}\\ {I}_{2}^{\lambda 1} & {I}_{2}^{\lambda 3}\end{array} & \begin{array}{cc}\cdots  & {I}_{1}^{\lambda 860}\\ \cdots  & {I}_{2}^{\lambda 860}\end{array}\\ \begin{array}{cc}{I}_{3}^{\lambda 1} & {I}_{3}^{\lambda 2}\\ {I}_{4}^{\lambda 1} & {I}_{4}^{\lambda 2}\end{array} & \begin{array}{cc}\cdots  & {I}_{3}^{\lambda 860}\\ \ldots  & {I}_{4}^{\lambda 860}\end{array}\end{array}]$$

The logarithm transform of column vector ${[{I}_{1}^{\lambda k},{I}_{2}^{\lambda k},{I}_{3}^{\lambda k},{I}_{4}^{\lambda k}]}^{T}$ of array (4) and the distance column vector ${[4.20,3.80,3.40,3.00]}^{T}$ were fitted to Eq. (5) through the least squares curve fitting method. The dependent variable *y* denotes the logarithm transform of the light intensity count, and the independent variable *x* denotes the distance. Slope *a* and intercept *b* were obtained through the fitting.

5 $$y=ax+b$$

The values of *a* for each wavelength were calculated and used to form a slope vector as described by Eq. (6).

6 $${\boldsymbol{s}}=[\begin{array}{cc}\begin{array}{cc}{a}^{\lambda 1} & {a}^{\lambda 2}\end{array} & \begin{array}{cc}\cdots  & {a}^{\lambda 1201}\end{array}\end{array}]$$

For convenience, symbols were used to represent the datasets involved in data processing and are as follows:

**A1:** The transmission spectrum obtained for a 3.0 mm total thickness of the blood bag and contained blood in Measurement 1.

**A2:** The transmission spectrum obtained for a 3.0 mm total thickness of the blood bag and contained blood in Measurement 2.

**S1**: The slope spectrum obtained in Measurement 1.

**S2**: The slope spectrum obtained in Measurement 2.

PLSR was used to establish a multivariate model relating the optical signal and the concentration. Among the 60 samples, 40 samples were used as a calibration set to develop a calibration model, and the remaining 20 samples were used as a prediction set to evaluate the performance of the model. Information about these samples are listed in Table 3. To enable a comparison, two different models were built using the optical data in Measurement 1. The datasets used in the modeling process are listed in Table 4. The performances of both models were assessed by using the determination coefficient for the calibration set (Rc), the determination coefficient for prediction (Rp), the root mean square error of the calibration set (RMSEC), and the root mean square error of the prediction set (RMSEP).

**Table 3:** Information about the Samples in Calibration Set and Prediction.

Data Set	Maximum value	Minimum value	Mean Value	Standard deviation
Calibration Set	72 g/L	161 g/L	123.15 g/L	19.41 g/L
Prediction Set	83 g/L	154 g/L	124.15 g/L	17.56 g/L

**Table 4:** Dataset used for PLSR model.

	Independent Variable (**X**)	Dependent Variable (**Y**)
Model 1	S1	Concentration of the Hb
Model 2	A1	Concentration of the Hb

To show the responses of the models to changes in blood bag thickness and blood thickness, datasets obtained in Measurement 2 were used to calculate Hb concentrations in both models.

## Results and Discussion

**Figure 7:**
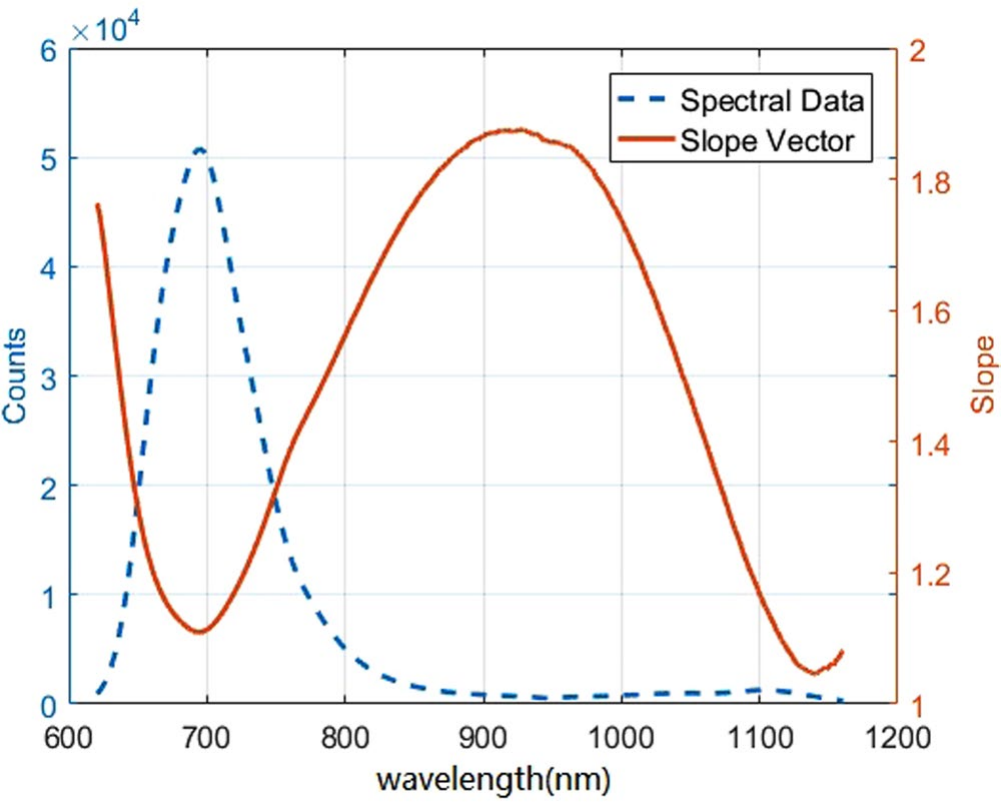
Spectral Data and slope vector of sample 1 in wavelength range 620–1170 nm.

**Figure 8:**
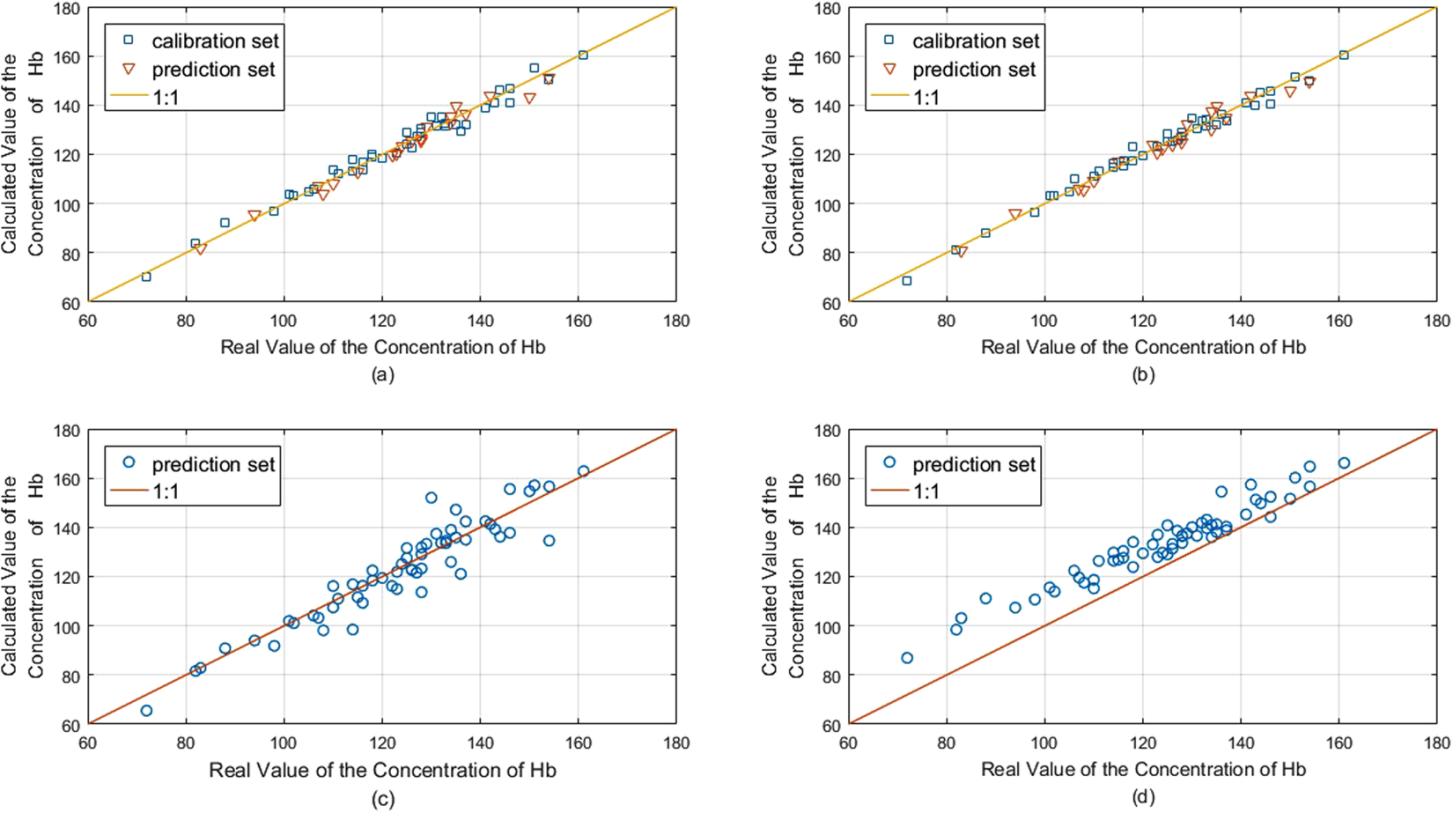
(**a**) Calculated Hb concentration in Model 1 using dataset S1. (**b**) Calculated Hb concentration in Model 2 using dataset A1. (**c**) Calculated Hb concentration by applying dataset S2 to Model 1. (**d**) Calculated Hb concentration by applying dataset A2 to Model 2.

The transmission spectrum and the slope vector of sample 1 at 4.2 mm between 620 and 1170 nm is displayed in Fig. 7. The modeling results corresponding to Model 1 are shown in Fig. 8(a). These results show that the calculated hemoglobin concentrations were close to the 1:1 best fit line in the calibration and prediction sets. The Rc, RMSEC, and RMSEP were calculated to evaluate the performance of the model. The results are shown in Table 5.

**Table 5:** Performance indictor of the two models.

	Dataset	Rc	Rp	RMSEC	RMSEP
Model 1	S1	0.9817	0.9702	2.29 g/L	2.78 g/L
	S2	—	0.87	—	10.16 g/L
Model 2	A1	0.9821	0.9722	2.30 g/L	2.75 g/L
	A2	—	0.65	—	17.22 g/L

The modeling results corresponding to Model 2 are shown in Fig. 8(b). The calculated hemoglobin concentrations were also close to the 1:1 best fit line. The Rc, Rp, RMSEC and RMSEP of Model 2 are shown in Table 5. Both models performed well in predicting hemoglobin concentrations. In clinical applications, the desired relative measurement error should be less than 3% for hemoglobin concentrations above 70 g. More work should be done to improve measurement accuracy.

Model 1 and Model 2 were built with the data collected in Measurement 1 at a blood bag wall thickness of approximately 0.5 mm. In Measurement 2, the blood bag wall was thicker than that in Measurement 1. We calculated hemoglobin concentrations by applying the data in Measurement 2 (denoted by S2 and A2) to Model 1 and Model 2. The results are shown in Fig. 8(c) and (d). The results were distorted in both models. The results for Model 2 showed greater distortion than those for Model 1. The root mean square error of the calculated hemoglobin concentration obtained using Model 1 was 10.16 g/L with a determination coefficient of approximately 0.87, whereas that obtained using Model 2 was 17.22 g/L with a determination coefficient of approximately 0.65. The model built with the slope spectrum was more robust and capable of adapting to changing blood bag wall thicknesses than the model built with the transmission spectrum with a single blood thickness.

## Conclusion

This study describes a method for determining hemoglobin levels in blood in blood bags without damaging the blood bag. The method involves a multi-pathlength spectrum detection device and a method of data processing. During data processing, the slope of the curve of the logarithmic light intensity and the optical pathlength is obtained by the curve fitting method, and a model between the slope and the hemoglobin concentration is established. This reduces measurement errors originating from differences in packaging thickness. The correlation coefficient of predicted hemoglobin levels in 20 samples was over 0.96, and the prediction error was less than 3 g/L. The experimental results demonstrate that our method can be used for the rapid, non-destructive detection of hemoglobin levels in red blood cell suspensions in blood bags.
